# Genome-Wide Identification and Characterization of *GASA* Gene Family in *Nicotiana tabacum*

**DOI:** 10.3389/fgene.2021.768942

**Published:** 2022-02-01

**Authors:** Zhaowu Li, Junping Gao, Genhong Wang, Shuaibin Wang, Kai Chen, Wenxuan Pu, Yaofu Wang, Qingyou Xia, Xiaorong Fan

**Affiliations:** ^1^ Tobacco Research Institute of Technology Centre, China Tobacco Hunan Industrial Corporation, Changsha, China; ^2^ State Key Laboratory of Crop Genetics and Germplasm Enhancement, Nanjing Agricultural University, Nanjing, China; ^3^ MOA Key Laboratory of Plant Nutrition and Fertilization in Low-Middle Reaches of the Yangtze River, Nanjing Agricultural University, Nanjing, China; ^4^ Biological Science Research Center, Southwest University, Chongqing, China

**Keywords:** *GASA*, Nicotiana tabacum, expression analysis, phylogenetic analysis, *cis*-elements

## Abstract

The gibberellic acid stimulated Arabidopsis (*GASA*) gene family is critical for plant growth, development, and stress response. *GASA* gene family has been studied in various plant species, however, the *GASA* gene family in tobacco (*Nicotiana tabacum*) have not been characterized in detail. In this study, we identified 18 *GASA* genes in the tobacco genome, which were distributed to 13 chromosomes. All the proteins contained a conserved GASA domain and highly specific 12-cysteine residues at the C-terminus. Phylogenetic analysis divided the *NtGASA* genes into three well-conserved subfamilies. Synteny analysis suggested that tandem and segmental duplications played an important role in the expansion of the *NtGASA* gene family. *Cis*-elements analysis showed that *NtGASA* genes might influence different phytohormone and stress responses. Tissue expression analysis revealed that *NtGASA* genes displayed unique or distinct expression patterns in different tissues, suggesting their potential roles in plant growth and development. We also found that the expression of *NtGASA* genes were mostly regulated by abscisic and gibberellic acid, signifying their roles in the two phytohormone signaling pathways. Overall, these findings improve our understanding of *NtGASA* genes and provided useful information for further studies on their molecular functions.

## Introduction

The gibberellic acid stimulated Arabidopsis (*GASA*) gene family is widespread in monocotyledonous and dicotyledonous plant species (Nahirñak et al., 2012). It encodes a class of cysteine-rich peptides characterized by a signaling amino acid region at the N-terminus and a conserved domain with 12 cysteines at the C-terminus (Silverstein et al., 2007). Previous studies indicated that peptides with a mutated or missing GASA domain are non-functional (Sun et al., 2013).

The *GAST1* gene, which was first identified in tomato and characterized as a gibberellic acid (GA)-deficient (*gib1*) mutant gene (Shi et al., 1992). Subsequently, many GASA homologs were identified in *Arabidopsis* (*Arabidopsis thaliana*), rice (*Oryza sativa*), wheat (*Triticum aestivum*), grapevine (*Vitis vinifera* L.), and tomato (*Solanum lycopersicum*) (Taylor and Scheuring, 1994; Aubert et al., 1998; Furukawa et al., 2006; Zhang et al., 2017; Ahmad et al., 2020). *GASA* gene family play important roles in plant growth and development. In *Arabidopsis*, *AtGASA4* is involved in light signaling and promotes floral development, whereas overexpression of *AtGASA5* delays flowering by downregulating the expression of *LFY* and *FT* and upregulating the expression of *FLC* (Zhang et al., 2009). In petunia, *GASA* are involved in floral transition and shoot elongation (Ben-Nissan et al., 2004).

Most *GASA* genes are involved in GA signaling pathways. In soybean (*Glycine max*), *GmGASA32* is upregulated by GA and interacts with *GmCDC25* to control plant height (Chen et al., 2021). In *Gerbera corolla*, *GEG*, a GASA family member, is stimulated by the exogenous application of GA_3_ and regulates cell expansion (Kotilainen et al., 1999). In strawberry (*Fragaria×ananassa*), *FaGAST* genes are upregulated by the exogenous application of GA and affect fruit ripening (de la Fuente et al., 2006). Besides, the expression of *GASA* genes is increased by other phytohormones such as brassinosteroid (BR), salicylic acid (SA), abscisic acid (ABA), naphthalene acetic acid (NAA), and indole-3-acetic acid (IAA) (Mutasa-Göttgens and Hedden, 2009; Lee et al., 2015; Qu et al., 2016; Boonpa et al., 2018). In rice, *OsGSR1*, a *GASA* family member, influences the BR signaling networks by interacting with the BR synthetase DIM/DWF1 (Wang et al., 2009). In *Arabidopsis*, *AtGASA2*, *AtGASA5*, and *AtGASA14* are involved in ABA signaling and affect flower induction. *AtGASA6* is an integrator of GA, ABA, and glucose signaling and controls seed germination and cell elongation (Zhang and Wang, 2008; Zhong et al., 2015). In apple (*Malus domestica*), the expression of *MdGASA* are upregulated by GA and ABA applications during the flowering stage (Fan et al., 2017).

*GASA* gene family also involved in plant response to abiotic and biotic stresses. In *Arabidopsis*, overexpression of *AtGASA4* suppresses the accumulation of reactive oxygen species (ROS) and nitric oxide in wounded leaves (Rubinovich and Weiss, 2010). In transgenic *Arabidopsis* plants, overexpression of *GASA4* from common beech (*Fagus sylvatica*) improves tolerance to salt, ROS, and heat stress (Alonso-Ramírez et al., 2009), overexpression of *GsGASA1* from soybean inhibits root growth in low temperatures and upregulates the expression of *RGL2* and *RGL3* (Li et al., 2011). In tomato, *Snakin*-*1* and *Snakin*-*2*, two *GASA*-like genes, are active *in vitro* against various bacteria (i.e., *Clavibacter michiganensis* subsp. *Sepedonicus*) and fungi (i.e., *Fusarium solani* and *Botrytis cinerea*) by regulating the redox levels (Almasia et al., 2008; Balaji and Smart, 2012). In rubber (*Hevea brasiliensis*), *HbGASA* genes are upregulated upon inoculation with *Colletotrichum gloeosporioides* and are involved in innate immunity by regulating ROS accumulation (An et al., 2018). Therefore, *GASA* gene family is involved in numerous physiological and biological processes, displaying complex and diverse functions.

Tobacco (*Nicotiana tabacum* L.) is widely cultivated and has been used as a model plant for biological research. *GASA* genes are important in plant growth and development, however, the tobacco *GASA* gene family were not characterized previously. In this study, we identified *GASA* gene family in the tobacco genome with bioinformatics methods, and characterized their gene structure, phylogenetic relationships, protein motifs, chromosomal locations, syntenic regions, *cis*-acting elements, and expression patterns in different tissues. Our findings provide useful clues for further studies of *GASA* gene family in tobacco.

## Materials and Methods

### Plant Materials and Growth Conditions

The cultivar K326 was used to analyze the expression of *GASA* genes in tobacco. Seeds were germinated in a nursery tray, seedlings were grown in a greenhouse with a cycle of 14 h light at 28°C/10 h dark at 25°C and relative humidity at 50–60%. Different tissues (root, stem, leaf, axillary bud, and flower) were collected at the flowering stage to analyze *NtGASA* expression. For phytohormone treatments, 3-week-old seedlings were transferred to plates containing 10 μM ABA, 10 μM GA, 10 μM IAA, 10 μM SA, 50 μM methyl-jasmonate (MeJA), or 1% (v/v) dimethyl sulfoxide (control) and incubated for 5 h under the same photoperiod, temperature, and humidity conditions (Kretzschmar et al., 2012; Zhang et al., 2018).

### Genome-Wide Identification of *NtGASA Genes*

For *NtGASA* identification, 15 GASA sequences were obtained from the *Arabidopsis* database (TAIR; http://www.arabidopsis.org) and used as queries for BLAST search against the Solanaceae Genomics Network (https://solgenomics.net/). Subsequently, the Hidden Markov Model-based profile of the GASA domain PFAM 02704 was used to verify the presence of the complete GASA domain in NtGASA sequences. The non-redundant putative NtGASA sequences with a conserved GASA domain were used for further bioinformatics (phylogenetic relationships, chromosomal locations, *Cis*-regulatory elements, etc) and expression analysis.

### Physicochemical Properties, Phylogenetic Relationships, Gene Structure, and Conserved Motifs Analysis

The isoelectric point, number of amino acids, and molecular weight of NtGASA were predicted using the ExPASy tool (http://web.expasy.org/protparam/). The sequences of GASA from *Arabidopsis* (AtGASA), rice (OsGASA), grapevine (*Vitis vinifera*; VvGASA), and tobacco (NtGASA) were used to construct a phylogenetic tree using MEGA 7.0 with the neighbor-joining (NJ) method and a bootstrap test of 1,000 replicates (Supplementary Table S1) (Tamura et al., 2007). The exon/intron structure of each *NtGASA* genes was illustrated using the Gene Structure Display Server (http://gsds.cbi.pku.edu.cn). The conserved motifs of NtGASA proteins were analyzed using MEME 5.1.1 (http://meme-suite. org/tools/meme) (Bailey et al., 2006).

### Chromosomal Locations and Gene Duplications Analysis

To obtain the chromosomal locations of *NtGASA* genes, the DNA sequence of each gene was mapped using MG2C 2.0 (http://mg2c.iask.in/mg2c_v2.0/). Segmental and tandem duplicated gene pairs within the tobacco genome, as well as collinear gene pairs among the *Arabidopsis*, rice, grapevine, and tobacco genomes, were identified using MCScanX (Wang et al., 2012). The collinearity map was constructed using Circos (Krzywinski et al., 2009). The synonymous and non-synonymous substitution rates (Ks and Ka, respectively) were calculated using KaKs_Calculator 2.0 (Wang et al., 2010).

### Expression Analysis of *NtGASA* Genes

Plant samples were collected from root, flower, leaf, stem and axillary bud of tobacco at flowering stage, total RNA was isolated from frozen samples using Trizol reagent (TaKaRa, Kusatsu, Shiga, Japan), and cDNA synthesis was performed using the M-MLV reverse transcriptase Kit (Thermo Fisher Scientific, Waltham, MA, United States), according to the manufacturer’s instructions. Quantitative reverse-transcription (qRT)-PCR was carried out using the Bio-Rad CFX96 real-time system (Bio-Rad, Hercules, CA, United States) with SYBR Green Master Mix (Bio-Rad). The *NtGADPH* gene was used as the internal control for data normalization, and the relative expression levels of selected genes were calculated using the 2^−ΔΔCt^ method (Schmittgen and Livak, 2008). The primers used for qRT-PCR are listed in Supplementary Table S3.

### Prediction and Classification of *Cis*-Regulatory Elements

The 3 kb DNA sequence upstream of the start codon of *NtGASA* genes was examined for the presence of *cis*-regulatory elements. *Cis*-regulatory elements in the promoters of each *NtGASA* gene were analyzed using the PlantCARE database (http://bioinformatics.psb.ugent.be/webtools/plantcare/html/) and classified according to their regulatory functions.

## Results

### Physicochemical Properties and Localization of NtGASA

To identify the GASA genes in tobacco, we used 15 AtGASA sequences as queries for BLAST search, and identified 18 putative NtGASA based on amino acid similarities. As shown in Table 1, the total and coding sequence lengths of *NtGASA* genes were 186 to3,715 bp and 186 to 444 bp, respectively. The deduced NtGASA proteins varied from 61 to 147 amino acids with a molecular weight of 6.6–16.17 kDa, and the isoelectric point ranged from 6.66 to 9.75. Apart from these, the instability index for most of the proteins (77.8%) were more than 35. According to the Grand average of Hydropathicity (GRAVY), the NtGASA proteins were hydrophilic except for NtGASA3, NtGASA4, and NtGASA9. The amino acid content of NtGASA was conserved, cysteine, lysine, and leucine were predominant amino residues. Most NtGASA proteins were localized in the extracellular membrane, chloroplasts, and mitochondria. Detailed information about NtGASA physicochemical characteristics is presented in Table 2.

**TABLE 1:** Detailed information of *NtGASA* gene families.

Genes	Gene ID	Chromosome no.	Start site	End site	Gene length (bp)	CDS (bp)	ORF (aa)
*NtGASA1*	*Nitab4.5_0000283g0170.1*	6	185015472	185,017,514	2042	330	109
*NtGASA2*	Nitab4.5_0000980g0290.1	12	80,486,202	80,487,672	1,470	315	104
*NtGASA3*	Nitab4.5_0003382g0010.1	18	97,583,429	97,584,414	985	312	103
*NtGASA4*	*Nitab4.5_0002950g0060.1*	10	98,281,808	98,282,601	793	312	103
*NtGASA5*	*Nitab4.5_0000192g0090.1*	8	6,360,799	6,361,800	1,001	330	109
*NtGASA6*	*Nitab4.5_0000560g0200.1*	21	75,261,510	75,263,202	1,692	408	135
*NtGASA7*	*Nitab4.5_0000422g0020.1*	8	79,059,072	79,060,803	1731	345	114
*NtGASA8*	*Nitab4.5_0003382g0030.1*	4	76,130,839	76,131,662	823	420	139
*NtGASA9*	*Nitab4.5_0001286g0050.1*	15	123,285,077	123,288,792	3,715	444	147
*NtGASA10*	*Nitab4.5_0002978g0140.1*	1	185,584,313	185,584,660	347	258	85
*NtGASA11*	Nitab4.5_0007189g0060.1	1	185,522,855	185,523,422	567	204	67
*NtGASA12*	*Nitab4.5_0000201g0290.1*	17	19,561,051	19,562,463	1,412	279	92
*NtGASA13*	Nitab4.5_0002171g0120.1	16	153,314,979	153,316,235	1,256	342	113
*NtGASA14*	Nitab4.5_0004707g0070.1	2	55,454,226	55,454,602	376	267	88
*NtGASA15*	*Nitab4.5_0000210g0050.1*	21	112,128,696	112,129,397	701	267	88
*NtGASA16*	*Nitab4.5_0006450g0020.1*	6	175,245,938	175,246,683	745	270	89
*NtGASA17*	*Nitab4.5_0000284g0030.1*	4	139,328,031	139,328,217	186	186	61
*NtGASA18*	*Nitab4.5_0000604g0040.1*	14	103,199,992	103,200,505	513	273	90

The Gene ID were modified with regular form.

**TABLE 2:** Amino acid composition and physiochemical characteristics of NtGASA protein.

Proteins	MW	PI	Major amino acid%	Instability index	GRAVY	Localization predicted
NtGASA1	12.24	9.37	C(11.9), K(8.3), S(8.3)	41.72	−0.344	nucl, mito, cyto
NtGASA2	11.08	9.03	C(11.5), S(9.6), L(8.7)	40.02	−0.114	extr, mito
NtGASA3	11.15	8.65	C(11.7), L(9.7), A(9.7)	35.46	0.172	extr, mito, vacu
NtGASA4	11.06	9.01	C(11.7), A(11.7), L(8.7)	32.41	0.179	extr, mito, cyto
NtGASA5	11.93	9.23	C(11), S(10.1), L(8.3)	50.59	−0.206	extr,nucl, mito
NtGASA6	15.2	9.75	A(10.4), K(9.6), C(8.9)	56.46	−0.388	mito, nucl, cyto
NtGASA7	12.69	9.64	A(11.4),C(10.5), K(10.5)	47.83	−0.310	extr,nucl, mito
NtGASA8	15.24	8.27	P(9.4), C(8.6), L(8.6)	59.78	−0.115	extr, cyto, nucl
NtGASA9	16.17	9.36	L(11.6), C(8.2), K(7.5)	36.04	0.048	golgi, endo, extr
NtGASA10	9.3	7.99	C(16.5), P(10.6), T(8.2)	51.26	−0.445	mito, nucl, cyto
NtGASA11	7.46	6.66	C(19.4), S(10.4), N(7.5)	64.88	−0.421	nucl, mito, cyto
NtGASA12	10.44	9.14	K(14.1), C(13), P(8.7)	30.82	−0.326	extr, mito, cyto
NtGASA13	12.7	9.2	P(15), C(11.5), K(9.7)	55.48	−0.296	extr,nucl, mito, cyto
NtGASA14	9.69	8.92	C(14.8), K(12.5), S(8)	25.92	−0.226	nucl, mito, cyto
NtGASA15	9.75	9.05	K(13.6), C(13.6), L(8.7)	37.25	−0.161	nucl, mito, extr
NtGASA16	10.02	9.54	K(15.7), C(13.5), A(9)	7.29	−0.091	mito, nucl, extr
NtGASA17	6.6	9.23	C(16.7), K(13.3), S(10)	52.6	−0.630	extr,nucl, mito
NtGASA18	9.72	8.97	C(13.3), S(12.2), K(11.1)	50.02	−0.007	extr, mito, cyto

MW, molecular weight (kDa); pI, isoelectric point; GRAVY, grand average of hydropathicity; A, Ala; C, Cys; L, Leu; K, Lys; P, Pro; S, Ser; T, Thr; Extra, extracellular; Vacu, vacuoles; Cyto, cytoplasm; Mito, mitochondria; Nucl, nucleus.

### Phylogenetic Analysis of *GASA* Genes From Tobacco, Rice, Grape, and *Arabidopsis*

**FIGURE 1:**
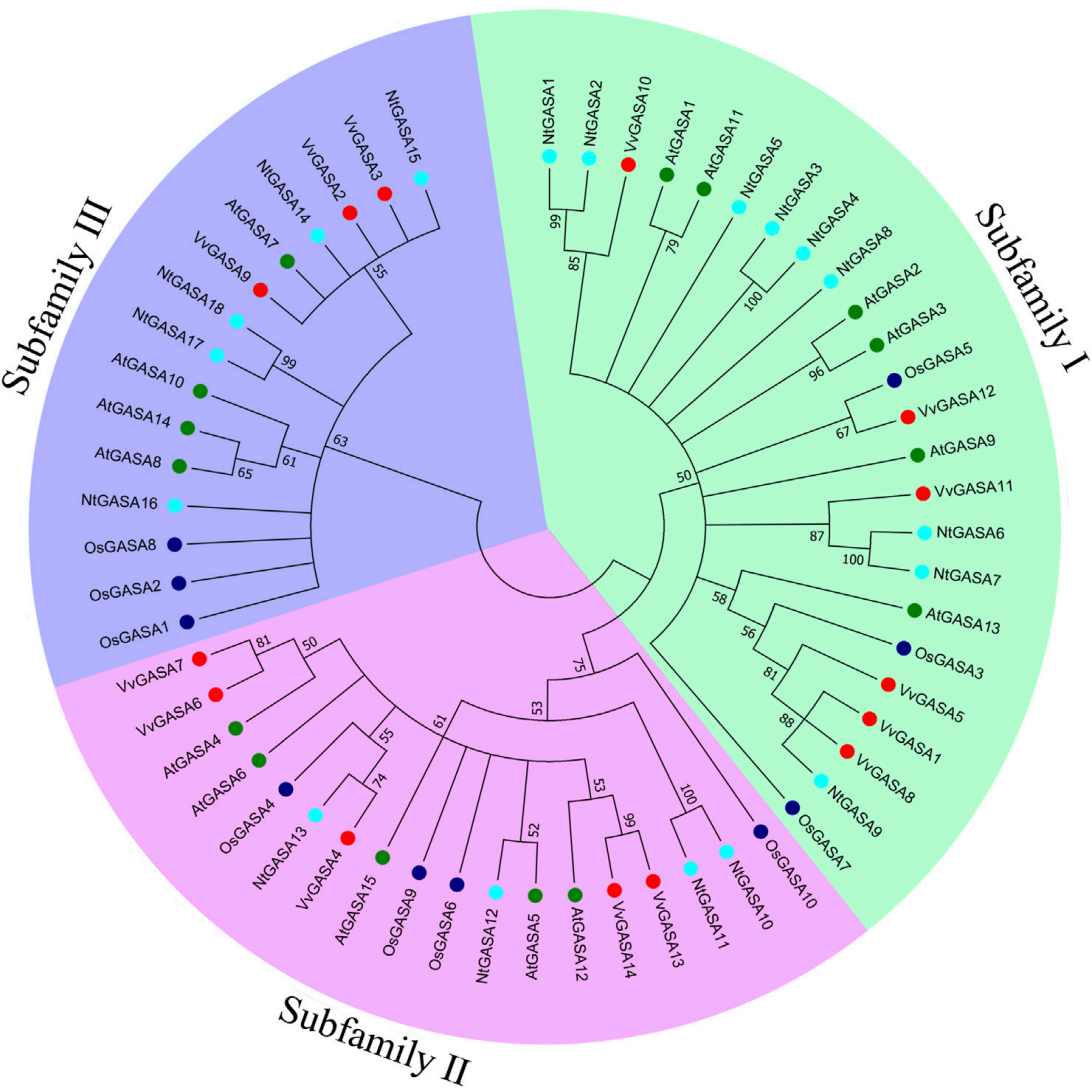
Phylogenetic analysis of GASA proteins from *Arabidopsis*, rice, grapevine and tobacco. A total of 15 GASA proteins from *Arabidopsis*, 10 GASA proteins from rice, 14 GASA proteins from grapevine, and 18 NtGASA proteins from tobacco were used to generate the unrooted neighbor-joining (NJ) tree with 1,000 bootstrap replicates. The GASA proteins are classified into three subfamilies (marked as I, II, III), and distinguished by different colors: AtGASA labeled in green, OsGASA labeled in blue, VvGASA labeled in red, and NtGASA labeled in cyan.

To characterize the phylogenetic relationships among *GASA* genes from *Arabidopsis*, rice, grapevine and tobacco, an unrooted NJ tree was constructed aligning 15 *AtGASA*, 10 *OsGASA*, 14 *VvGASA*, and 18 *NtGASA*. According to the phylogenetic tree, *GASA* genes could be classified into three subfamilies: subfamily I included six *AtGASA* genes (*AtGASA1*/*2*/*3*/*9*/*11/13*), three *OsGASA* genes (*OsGASA3*/*5*/*7*), six *VvGASA* genes (*VvGASA1*/*5*/*8*/*10*/*11*/*12*), and nine *NtGASA* genes (*NtGASA1*–*9*). Subfamily II included five *AtGASA* genes (*AtGASA4*/*5*/*6*/*12*/*15*), four *OsGASA* genes (*OsGASA4/6*/*9/10*), five *VvGASA* genes (*VvGASA4*/*6*/*7*/*13*/*14*), and four *NtGASA* genes (*NtGASA10*–*13*). Subfamily III included four *AtGASA* genes (*AtGASA7*/*8*/*10*/*14*), three *OsGASA* genes (*OsGASA1*/*2*/*8*), three *VvGASA* genes (*VvGASA2*/*3/9*), and five *NtGASA* genes (*NtGASA14*–*18*) (Figure 1). Therefore, subfamily I had more GASA members from *Arabidopsis*, grapevine, and tobacco, whereas subfamily II had more GASA members from rice.

### Chromosomal Distributions and Synteny Analysis of *NtGASA* Genes

**FIGURE 2:**
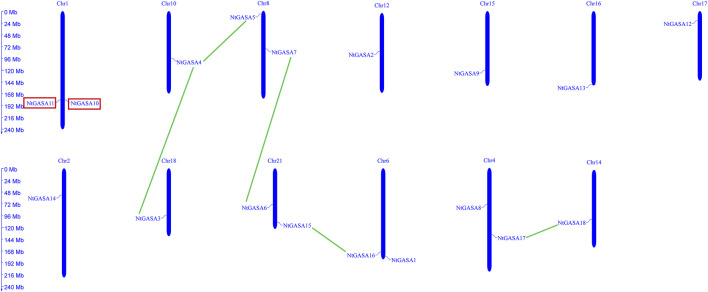
Chromosomal distributions and gene duplication of *NtGASA* genes. Chromosome size is indicated by its relative length. Segmental duplicated *NtGASA* genes are connected by green colored lines, and red box shows two tandem duplicated gene pairs.

The localizations of the *NtGASA* genes in the chromosomes of tobacco were further determined. Using a simplified physical map, we found that the 18 *NtGASA* genes were unevenly distributed in 11 chromosomes in the tobacco genome. Chromosome (Chr.) 1, 4, 6, 8, and 21 contained two copies each, whereas Chr. 2, 10, 12, 14, 15, 16, 17, and 18 contained one copy each (Figure 2).

Tandem and segmental duplicates play an important role in the expansion of gene families. Two genes (*NtGASA10* and *NtGASA11*) were tandemly duplicated on Chr.1. In addition, five pairs (*NtGASA3/NtGASA4*, *NtGASA4/NtGASA5*, *NtGASA6/NtGASA7*, *NtGASA15/NtGASA16*, and *NtGASA17/NtGASA18*) were segmental duplicated (Figure 2). All tandem and segmental duplicates had Ka/Ks values less than 1 (Table 3), indicating that the six gene pairs evolved under the influence of purifying selection.

**TABLE 3:** Calculation of Ka and Ks ratios of six duplicated *NtGASA* gene pairs.

Gene 1	Gene 2	Ka	Ks	Ka/Ks	Gene Duplications
*NtGASA3*	*NtGASA4*	0.0239	0.1033	0.2322	Segmental
*NtGASA4*	*NtGASA5*	0.1907	0.4244	0.4495	Segmental
*NtGASA6*	*NtGASA7*	0.0187	0.0214	0.8724	Segmental
*NtGASA15*	*NtGASA16*	0.1112	0.2546	0.4369	Segmental
*NtGASA17*	*NtGASA18*	0.2699	0.5933	0.4549	Segmental
*NtGASA10*	*NtGASA11*	0.0442	0.1293	0.3423	Tandem

**FIGURE 3:**
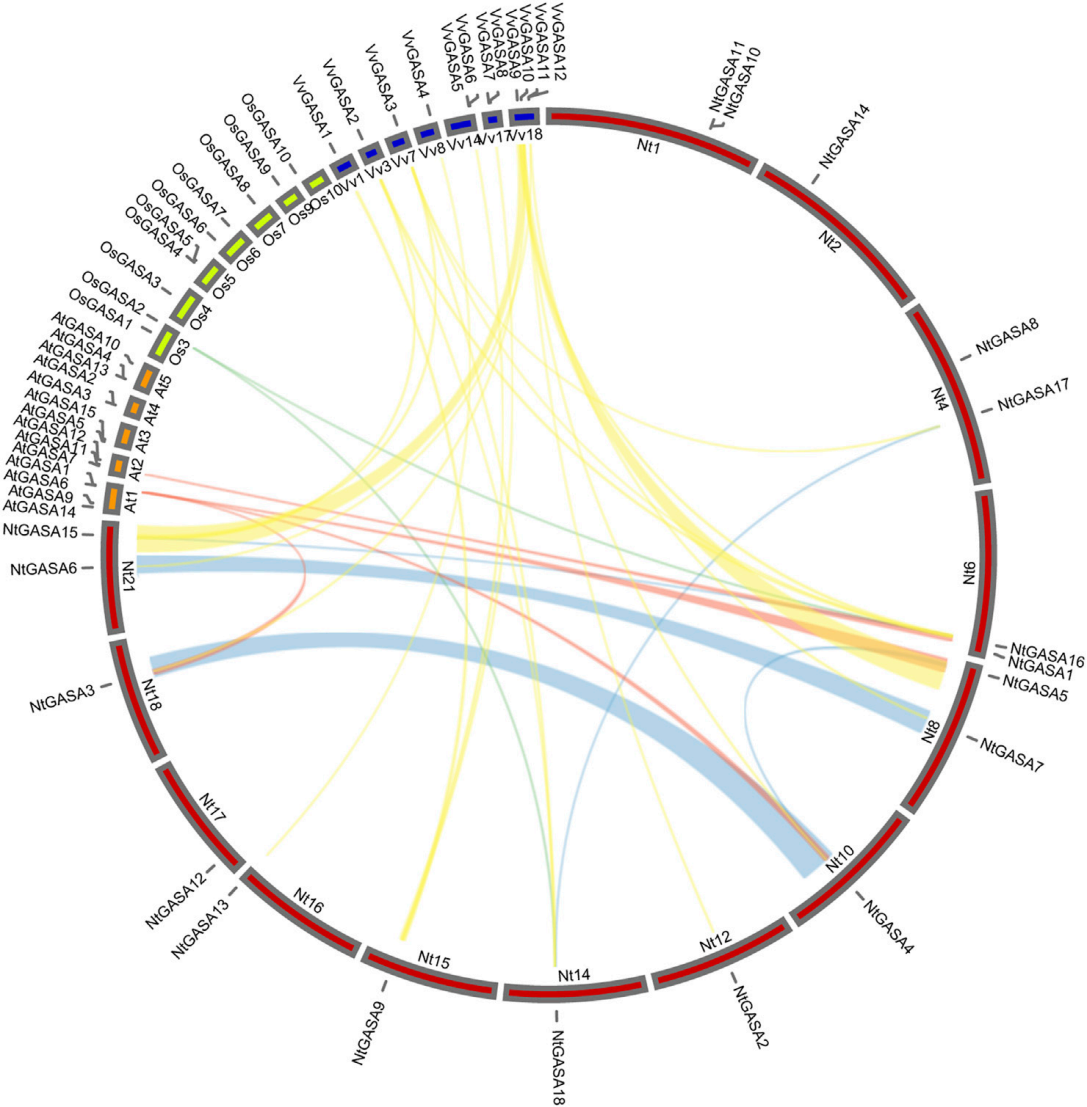
Collinear analysis of *GASA* genes between tobacco (Nt), grapevine (Vv), rice (Os), and *Arabidopsis* (At). Yellow, red and cyan lines represent the collinear gene pairs between tobacco and grapevine, tobacco and *Arabidopsis*, tobacco and rice chromosomes, respectively. Blue lines indicate the segmental duplicated *NtGASA* genes.

We constructed a collinearity plot of the tobacco, rice, grapevine and *Arabidopsis GASA* gene families to further explore the evolutionary relationships among *GASA* genes from different species. A total of 2, 4, and 19 collinear gene pairs were identified between tobacco and rice, tobacco and *Arabidopsis*, and tobacco and grapevine, respectively. Most collinear relationships were many-to-one matches, such as (*NtGASA3*, *NtGASA4*, *NtGASA5*)/*AtGASA1*, (*NtGASA16*, *NtGASA18*)/*OsGASA2*, (*NtGASA2*, *NtGASA3*, *NtGASA4*, *NtGASA5*)/*VvGASA10* and (*NtGASA6*, *NtGASA7*)/*VvGASA11*. There were also one-to-many matches, such as *NtGASA*9/(*VvGASA1*, *VvGASA5*, *VvGASA8*), *NtGASA15*(*VvGASA2*, *VvGASA3*, *VvGASA9*)*,NtGASA16*/(*VvGASA2*, *VvGASA3*, *VvGASA9*) and *NtGASA18/*(*VvGASA2*, *VvGASA3*). The one-to-one matches were *NtGASA16*/*AtGASA7,NtGA*SA*13*/*VvGASA4* and *NtGA*SA*17*/*VvGASA3* (Figure 3, Supplementary Table S2). These results indicate that *GASA* genes were relatively conserved between different species and might originate from the same ancestor.

### Analysis of Conserved Motifs and Gene Structure

**FIGURE 4:**
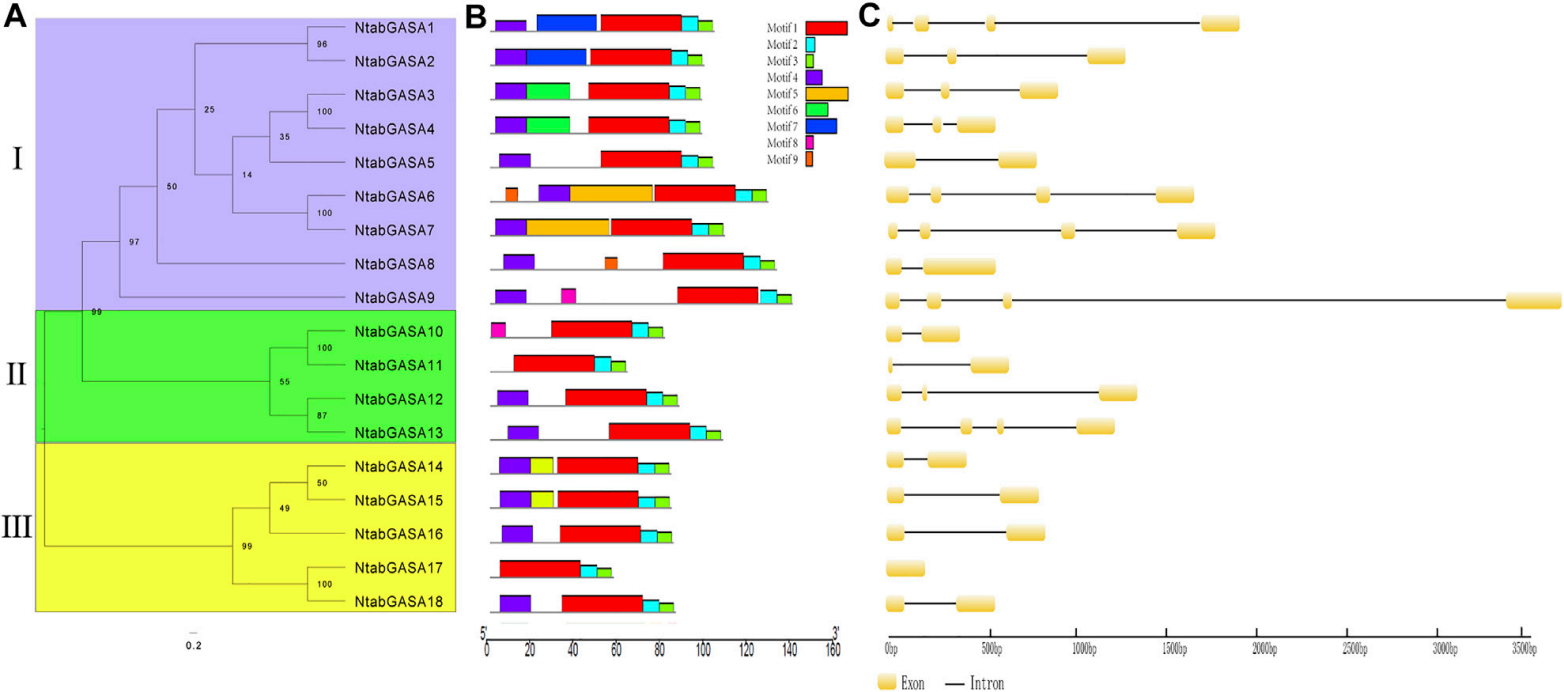
Phylogenetic relationship, conserved motifs of NtGASA proteins and exon–intron structures of *NtGASA* genes. **(A)** An unrooted phylogenetic tree constructed based on NtGASA protein sequences, different colors indicate different subgroups. **(B)** Conserved motifs in the NtGASA proteins. The conserved motifs were identified using MEME with complete protein sequences. Different motifs were displayed by various colors. **(C)** Exon–intron distribution of *NtGASA* genes.

To further explore the phylogenetic relationships among *NtGASA* genes, an unrooted tree was constructed between *NtGASA* genes. In concordance with the phylogenetic tree including the tobacco, *Arabidopsis*, grapevine, and rice *GASA* genes, this analysis also supported the classification of *NtGASA* genes into three subfamilies (Figure 4A). The number of conserved motifs in NtGASA proteins varied from three to 6 (Figure 4B). The highly conserved motifs 1, 2, and three were detected in all 18 NtGASA proteins, whereas motif five was only found in NtGASA6 and NtGASA7, motif eight were only found in NtGASA9 and NtGASA10. The diversity of motifs in different subfamilies suggests that NtGASA functions have tended to diversify during evolution.

Structural analysis revealed that the length, arrangement, and position of introns in *NtGASA* genes were relatively less conserved. For instance, subfamilies I and II contained one to three introns and subfamily III contained one intron, except for *NtGASA8* that had only one exon and no intron (Figure 4C). Intron gain and loss is a frequent phenomenon during evolution and can increase the complexity of gene structures.

**FIGURE 5:**
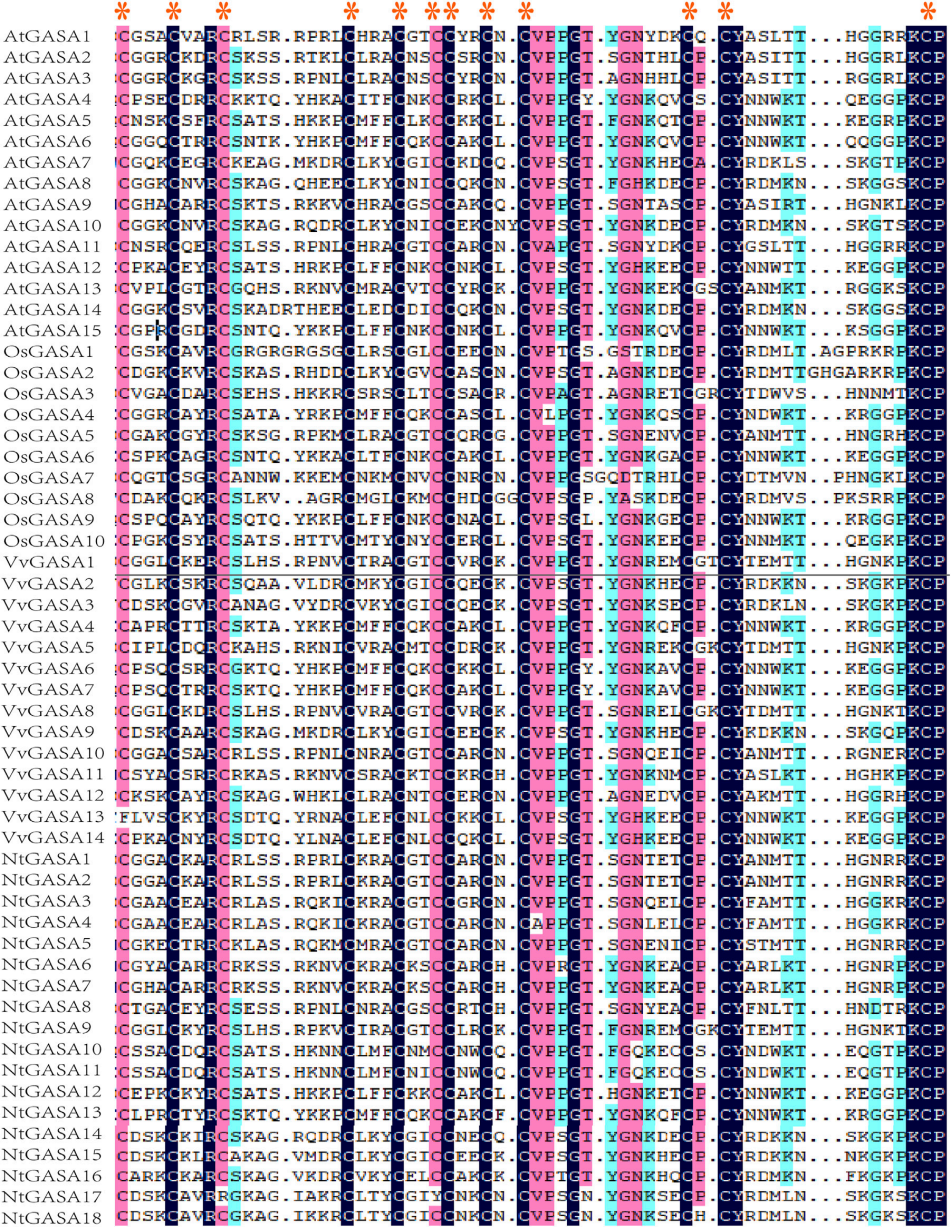
Alignment of the GASA domain from AtGASA, OsGASA, VvGASA, and NtGASA proteins, red asterisk represented their conserved cysteines.

In previous findings, putative GASA protein possesses highly conserved C-terminal domain that containing 12 conserved cysteines (Aubert et al., 1998). Amino-acid sequence comparison of AtGASA, OsGASA, VvGASA, and NtGASA revealed that all putative NtGASA proteins shared a conserved GASA domain, except for NtGASA17, in which GASA domains were mutated by the insertion of several amino acids (Figure 5).

### Tissue-Specific Expression Profiling of *NtGASA* Genes

**FIGURE 6:**
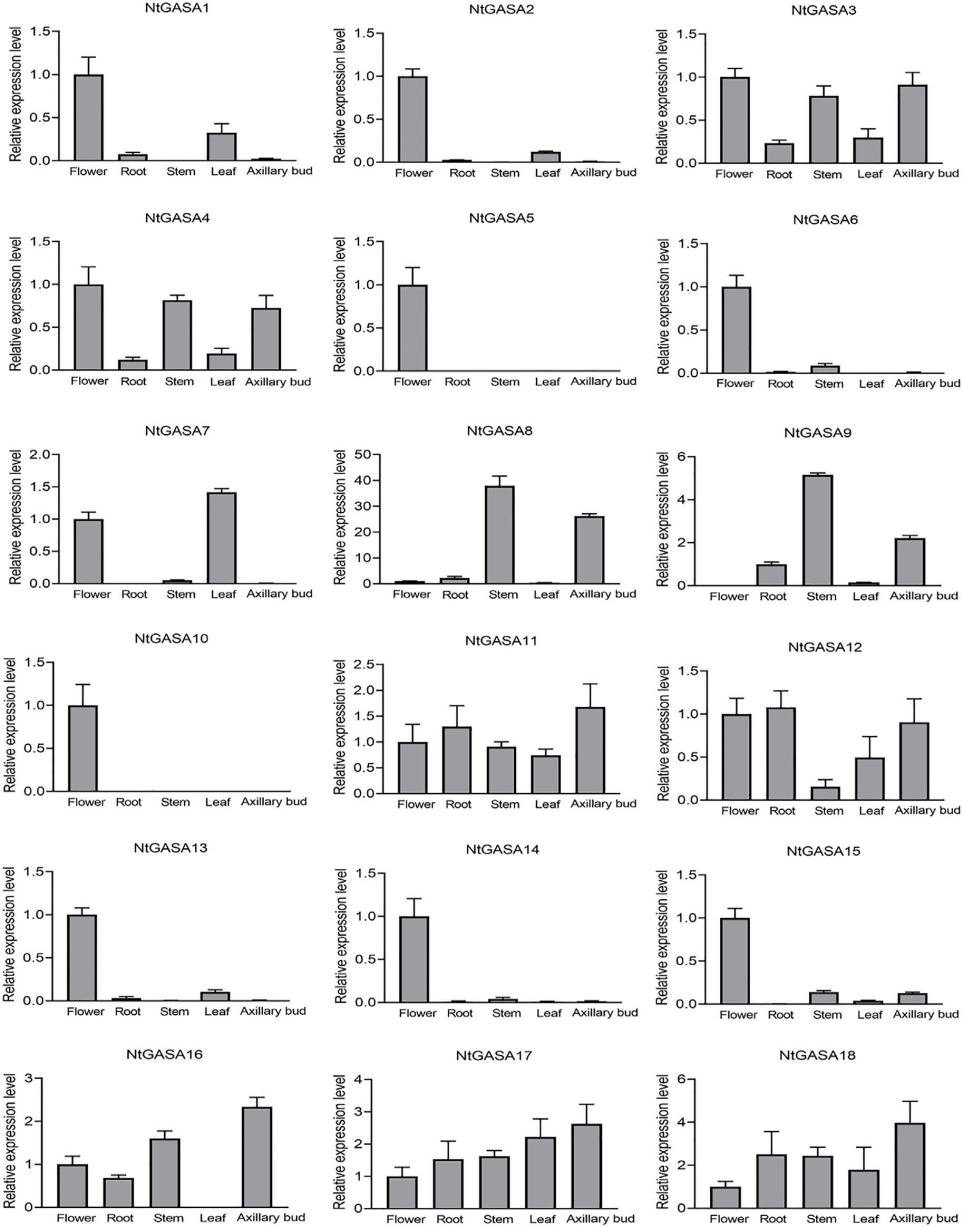
Expression patterns of *NtGASA* genes in different tissues and organs. Transcript levels in flowers were set to one, in *NtGASA9*, transcript levels in root was set to one. Each value represents the mean ± standard error of three biological replicates.

The spatio-temporal expression analysis of genes can provide information about gene function. We performed qRT-PCR for expression profiling of the *NtGASA* genes in the root, flower, leaf, stem, and axillary bud. The expression profiling showed that most *NtGASA* genes had diverse expression patterns in different tissues. *NtGASA3*, *NtGASA11*, *NtGASA17*, and *NtGASA18* were expressed relatively ubiquitously. Whereas many *NtGASA* genes showed high expression in specific tissues, such as *NtGASA9* had the highest expression levels in the stem, *NtGASA7* in the leaf, *NtGASA16* in the axillary bud, and *NtGASA2*, *NtGASA5*, *NtGASA6*, *NtGASA10*, *NtGASA13*, *NtGASA14,* and *NtGASA15* in the flower. Notably, *NtGASA12* had the lowest expression levels in the stem. In general, most *NtGASA* genes were highly expressed in reproductive organs (i.e., flower) compared with vegetative parts (i.e., leaf and stem) (Figure 6).

### Analysis of *Cis*-Elements in the Promoters of *NtGASA* Genes

**FIGURE 7:**
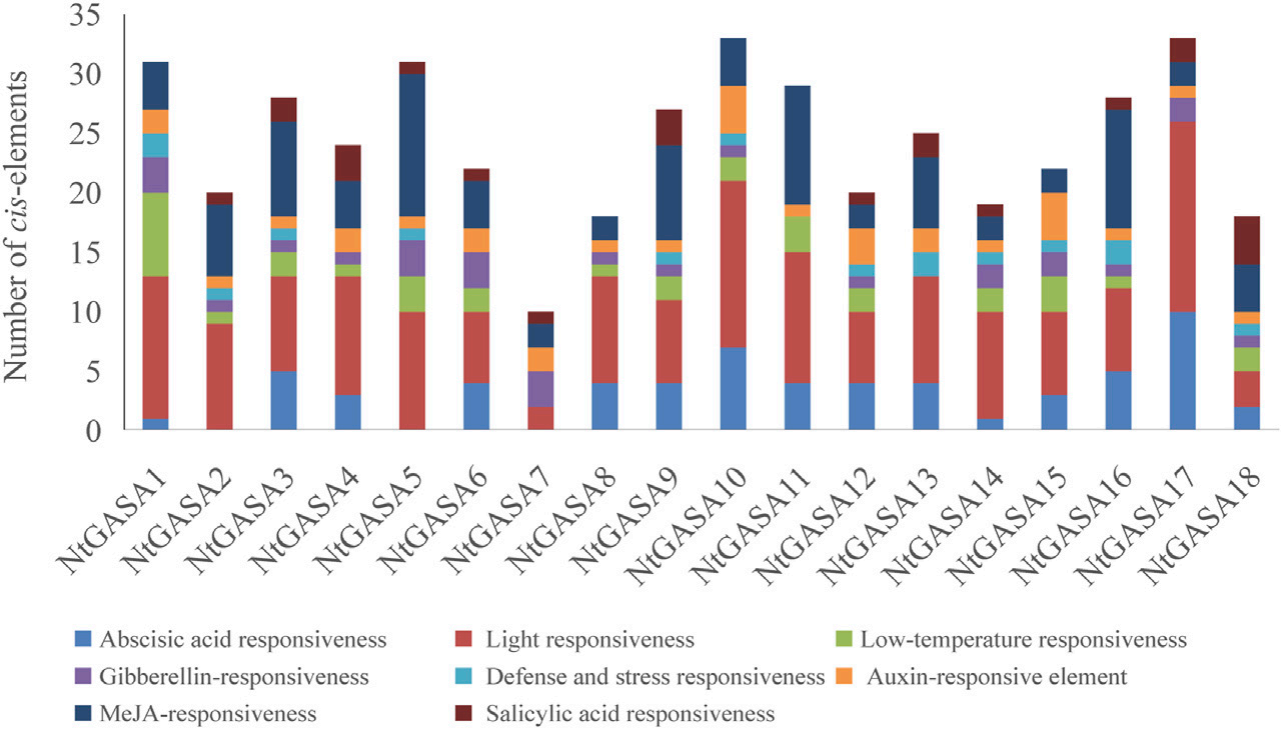
*Cis*-element analysis in the *NtGASA* promoters.

The study of *cis*-elements could provide clues about regulatory pathways of gene expression, then we analyzed the 3,000-bp upstream promoter sequences of *NtGASA* genes. The largest number of *cis*-elements observed across the *NtGASA* genes was associated with light-responsiveness. In addition, *cis*-elements involved in phytohormone (i.e., ABA, GA, IAA, SA, and MeJA) and stress (i.e., low temperature) responses were also identified in the promoter sequences of *NtGASA* genes (Figure 7). The diversity in response elements indicated the regulatory roles of *NtGASA* genes in various physiological and biological processes.

### Expression Profiling of *NtGASA* Genes Under Various Phytohormone Treatments

**FIGURE 8:**
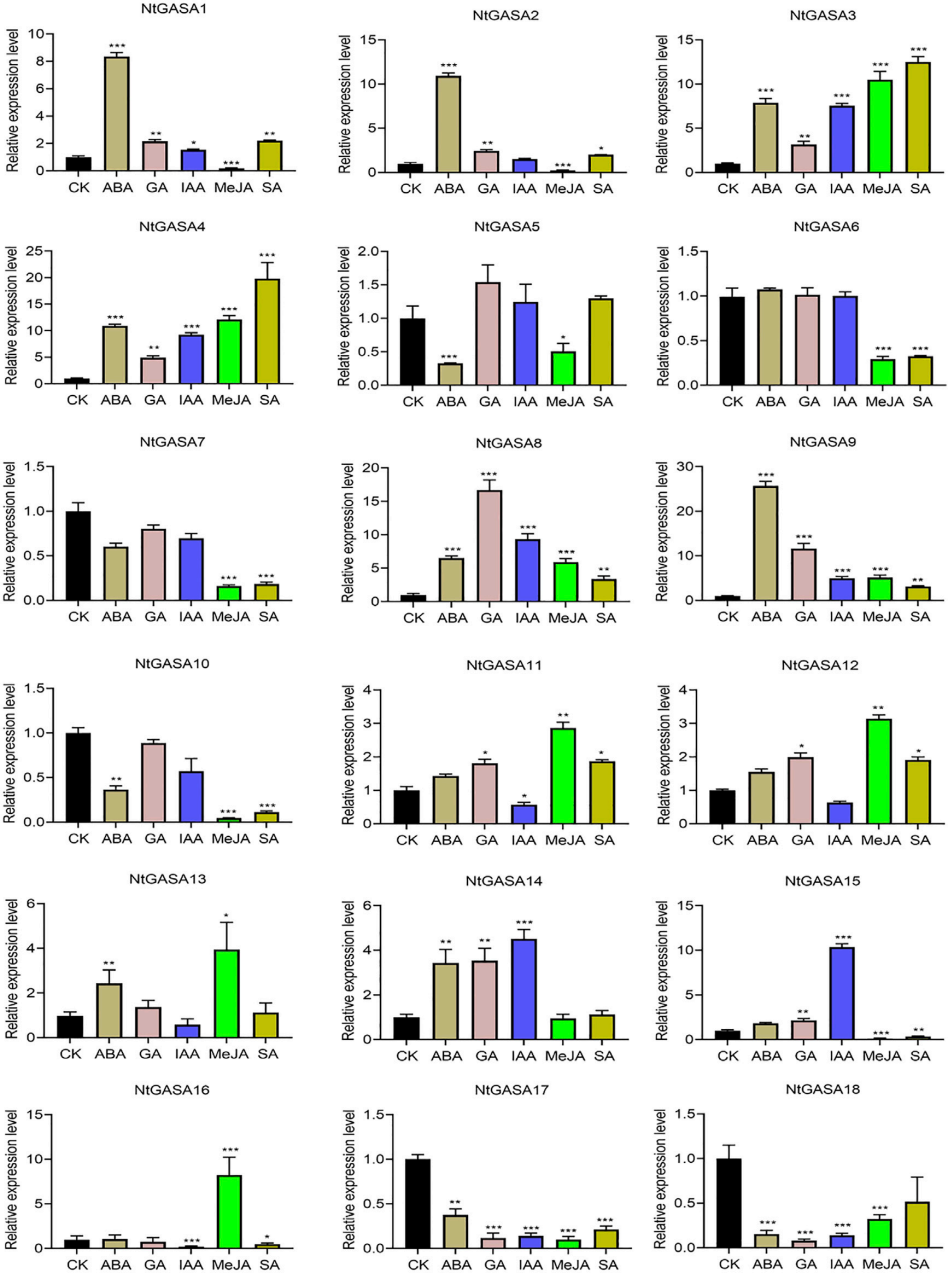
Expression patterns of *NtGASA* genes under various phytohormone treatments. 3-week-old seedlings treated with 10 μM ABA, 10 μM GA, 10 μM IAA, 10 μM SA, and 50 μM MeJA were collected for expression analysis, seedlings treated with 1% (v/v) DMSO served as controls. Each value represents the mean ± standard error of three biological replicates. Asterisks denote significant differences between the hormone treatment and control sample. (Student’s *t*-test, *p < 0.05, **p < 0.01, ***p < 0.001).

The results of the *cis*-element analysis indicated that *NtGASA* genes might be related to many plant hormone responses. To elucidate the expression pattern of *NtGASA* genes and their possible roles in phytohormone signaling pathway, the transcript levels of all *NtGASA* genes under ABA, GA, IAA, MeJA, SA treatment were investigated. The expression profiling of *NtGASA* under different phytohormone treatments showed diverse patterns compared with the control. For instance, ABA significantly upregulated the expression of *NtGASA1/2/3/4/8/9/13/14*, but inhibited the expression of *NtGASA5/10/17/18*. Most of the *NtGASA* genes were highly expressed by GA treatment, except for *NtGASA6/7/10/16/17/18*. After IAA treatment, the expression of *NtGASA1/3/4/8/9/14/15* were significantly upregulated, *NtGASA11* was downregulated. The expression of *NtGASA1/2/3/4/8/9/11/12* were significantly upregulated by SA treantment, *NtGASA6/7/10/15/16* were downregulated. Moreover, after MeJA treantment, the expressions of *NtGASA3/4/8/9/11/12/13/16* were significantly upregulated, and the expression of *NtGASA16* was only upregulated by MeJA treatment, *NtGASA1/2/5/6/7/15* were downregulated*.* Interestingly, the expression of *NtGASA17* and *NtGASA18* were downregulated by all phytohormones (Figure 8). These findings indicated that different *NtGASA* genes might play distinctive roles in response to various phytohormone signals.

## Discussion

GASA influence various biological processes and signal transduction pathways, and then playing critical roles in plant growth and development (Choi et al., 2017). Due to complexities in functional mechanisms, different members of the *GASA* gene family have identical or diverse functions during the vegetative and reproductive stages. In *Arabidopsis*, *AtGASA5* is activated by ABA during seed dormancy, whereas *AtGASA4* is expressed during germination (Zhang et al., 2009). In strawberry, *FaGAST1* and *FaGAST2* have distinct expression patterns and belong to different subfamilies, but they are both involved in similar physiological functions and synergistically affect the fruit cell size (Moyano-Cañete et al., 2013). The *GASA* gene family is found in many plant species, but little is known about the corresponding genes in tobacco. Here, we conducted a comprehensive genome-wide identification and expression profiling study of *GASA* gene family in tobacco.

We identified 18 *NtGASA* genes in the tobacco genome, more than those previously found in *Arabidopsis*, rice, grapevine, potato, and soybean (Roxrud et al., 2007; Nahirñak et al., 2016; Ahmad et al., 2019; Muhammad et al., 2019; Ahmad et al., 2020). Based on phylogenetic analyses, the identified *NtGASA* genes were divided into three subfamilies, of which subfamily I contained the highest number of genes (Figure 1). Physicochemical analysis showed that all the identified NtGASA had low molecular weight and were alkaline, except for NtGASA11 (Table 2), consistently with previously reported results in *Arabidopsis*, grapevine, and apple (Herzog et al., 1995; Berrocal-Lobo et al., 2002). In addition, cysteine was the predominant amino acid among NtGASA proteins, probably due to the highly conserved 12-cysteine residue at the C-terminus (Table 2; Figure 5).

We also found that motif 1, 2, and three were highly conserved and present in all 18 NtGASA proteins, whereas motif five and eight were only present in NtGASA6/7 and NtGASA9/10, respectively (Figure 4B). Variation in conserved motifs suggested that NtGASA functions were diversified during evolution. Indeed, *NtGASA* gene structure analysis revealed that the number of introns was varied from 0 to 3 (Figure 4C), indicating that a gain and loss of introns occurred over time, which may be caused by chromosomal rearrangements (Xu et al., 2012; Guo et al., 2013).

Tandem or segmental duplication, as well as whole-genome duplication, markedly affect the evolution of gene families (Vision Todd et al., 2000; Paterson et al., 2010). Our results showed that the presence of both tandem and segmental duplications contributed to the evolutionary process of *NtGASA* genes. We identified one pair of tandem duplicated *NtGASA* genes and five pairs of segmental duplicated *NtGASA* genes throughout the genome (Table 3), these results corroborates the previous findings that segmental duplications occur more frequently than tandem duplications (Zhang et al., 2020). The collinear analysis of *GASA* genes from *Arabidopsis*, rice, grapevine, and tobacco showed that the existence of more collinear gene pairs between grapevine and tobacco (Figure 3), suggesting a closer evolutionary distance between the two plant species.

We further analyzed the expression profiles of *NtGASA* genes in different tissues and found a large variety of expression patterns. Several genes (i.e., *NtGASA11* and *NtGASA17*) showed ubiquitous expression, whereas most *NtGASA* genes were upregulated only in specific tissues (i.e., *NtGASA9* in the stem; *NtGASA7* in the leaf; *NtGASA16* in the axillary bud; and *NtGASA2/5/6/10/13/14/15* in the flower) (Figure 6). Previous studies indicated that *GASA* genes contribute to the regulation of flower induction in various species such as *Petunia hybrida*, *Gerbera hybrida*, rice, and cotton (Ben-Nissan et al., 2004; Peng et al., 2010; Muhammad et al., 2019; Qiao et al., 2021). Here, 13 *NtGASA* genes showed high expression in the flower, suggesting that they might play important roles in floral development.

The promoter region of a gene is related to its function, and thus, the analysis of *cis*-elements assists in its functional characterization (Lescot et al., 2002). Our results showed that *NtGASA* genes contained various regulation elements on their promoters, such as *cis*-acting regulatory elements essential for light, phytohormone, and stress responses (Figure 7), suggesting their involvement in multiple signaling pathways. *GASA* transcripts are responsive to phytohormones and share common phytohormone-related *cis*-elements. In the present study, we found that all *NtGASA* genes were regulated by multiple phytohormones, especially ABA and GA, except for *NtGASA16*, that was only induced by MeJA. Besides, *NtGASA17* and *NtGASA18* were downregulated by all applied phytohormones (ABA, GA, IAA, SA, or MeJA), indicating that unidentified *cis*-elements might regulate their expression (Figure 8). The complex expression patterns of *NtGASA* genes under phytohormone applications highlighted their potential integral roles in various physiological processes.

## Conclusion

To our knowledge, this is the first report on the identification and characterization of *GASA* genes in tobacco. We identified 18 *NtGASA* genes and analyzed their physicochemical characteristics, phylogenetic relationships, gene structure, conserved motifs, chromosomal locations, synteny, and *cis*-elements in the promoters, which showed a clear evolutionary history for this family in tobacco. We also studied the expression patterns of *NtGASA* genes in various tissues and under different phytohormone applications. Overall, our results provided insights into the role of *NtGASA* genes in several physiological and biological pathways and laid a solid foundation for further exploring the underlying molecular and biochemical mechanisms.

## Data Availability

The original contributions presented in the study are included in the article/Supplementary Material, further inquiries can be directed to the corresponding authors.
