# Supplementary material for: Genome-Wide Identification and Characterization of GASA Gene Family in Nicotiana tabacum
Source: Front Genet. 2022 Feb 1;12:768942. doi: 10.3389/fgene.2021.768942 (PMC8844377; doi:10.3389/fgene.2021.768942)
Supplement: Supplementary file 1 [file Table1.docx]

Table S1 The GASA protein sequences of *Arabidopsis*, rice, grapevine, and tobacco

***Arabidopsis thaliana***

AtGASA1

MAISKALIASLLISLLVLQLVQADVENSQKKNGYAKKIDCGSACVARCRLSRRPRLCHRACGTCCYRCNCVPPGTYGNYDKCQCYASLTTHGGRRKCP

AtGASA2

MAVFRSTLVLLLIIVCLTTYELHVHAADGAKVGEGVVKIDCGGRCKDRCSKSSRTKLCLRACNSCCSRCNCVPPGTSGNTHLCPCYASITTHGGRLKCP

AtGASA3

MAIFRSTLVLLLILFCLTTFELHVHAAEDSQVGEGVVKIDCGGRCKGRCSKSSRPNLCLRACNSCCYRCNCVPPGTAGNHHLCPCYASITTRGGRLKCP

AtGASA4

MAKSYGAIFLLTLIVLFMLQTMVMASSGSNVKWSQKRYGPGSLKRTQCPSECDRRCKKTQYHKACITFCNKCCRKCLCVPPGYYGNKQVCSCYNNWKTQEGGPKCP

AtGASA5

MANCIRRNALFFLTLLFLLSVSNLVQAARGGGKLKPQQCNSKCSFRCSATSHKKPCMFFCLKCCKKCLCVPPGTFGNKQTCPCYNNWKTKEGRPKCP

AtGASA6

MAKLITSFLLLTILFTFVCLTMSKEAEYHPESYGPGSLKSYQCGGQCTRRCSNTKYHKPCMFFCQKCCAKCLCVPPGTYGNKQVCPCYNNWKTQQGGPKCP

AtGASA7

MKIIVSILVLASLLLISSSLASATISDAFGSGAVAPAPQSKDGPALEKWCGQKCEGRCKEAGMKDRCLKYCGICCKDCQCVPSGTYGNKHECACYRDKLSSKGTPKCP

AtGASA8

MKLVVVQFFIISLLLTSSFSVLSSADSSCGGKCNVRCSKAGQHEECLKYCNICCQKCNCVPSGTFGHKDECPCYRDMKNSKGGSKCP

AtGASA9

MKKMNVVAFVTLIISFLLLSQVLAELSSSSNNETSSVSQTNDENQTAAFKRTYHHRPRINCGHACARRCSKTSRKKVCHRACGSCCAKCQCVPPGTSGNTASCPCYASIRTHGNKLKCP

AtGASA10

MKFPAVKVLIISLLITSSLFILSTADSSPCGGKCNVRCSKAGRQDRCLKYCNICCEKCNYCVPSGTYGNKDECPCYRDMKNSKGTSKCP

AtGASA11

MAVFRVLLASLLISLLVLDFVHADMVTSNDAPKIDCNSRCQERCSLSSRPNLCHRACGTCCARCNCVAPGTSGNYDKCPCYGSLTTHGGRRKCP

AtGASA12

MMKLIVVFVISSLLFATQFSNGDELESQAQAPAIHKNGGEGSLKPEECPKACEYRCSATSHRKPCLFFCNKCCNKCLCVPSGTYGHKEECPCYNNWTTKEGGPKCP

AtGASA13

MALSLLSVFIFFHVFTNVVFAASNEESNALVSLPTPTLPSPSPATKPPSPALKPPTPSYKPPTLPTTPIKPPTTKPPVKPPTIPVTPVKPPVSTPPIKLPPVQPPTYKPPTPTVKPPSVQPPTYKPPTPTVKPPTTSPVKPPTTPPVQSPPVQPPTYKPPTSPVKPPTTTPPVKPPTTTPPVQPPTYNPPTTPVKPPTAPPVKPPTPPPVRTRIDCVPLCGTRCGQHSRKNVCMRACVTCCYRCKCVPPGTYGNKEKCGSCYANMKTRGGKSKCP

AtGASA14

MKMPVVVQFFIISLLLTSSFYVLSSADSSACGGKCSVRCSKADRTHEECLEDCDICCQKCNCVPSGTYGNKDECPCYRDMKNSKGGSKCP

AtGASA15

MATKLSIIVFSIVVLHLLLSAHMHFLINVCAECETKSAIPPLLECGPRCGDRCSNTQYKKPCLFFCNKCCNKCLCVPPGTYGNKQVCPCYNNWKTKSGGPKCP

***Oryza sativa***

OsGASA1

MKTRRAALLMLLLLVVVAAASWPQPCDAASGFCGSKCAVRCGRGRGRGSGCLRSCGLCCEECNCVPTGSGSTRDECPCYRDMLTAGPRKRPKCP

OsGASA2

MKLNTTTTLALLLLLLLASSSLQVSMAGSDFCDGKCKVRCSKASRHDDCLKYCGVCCASCNCVPSGTAGNKDECPCYRDMTTGHGARKRPKCP

OsGASA3

MAPGKLAVFALLASLLLLNTIKAADYPPAPPLGPPPHKIVDPGKDCVGACDARCSEHSHKKRCSRSCLTCCSACRCVPAGTAGNRETCGRCYTDWVSHNNMTKCP

OsGASA4

MEGVGVGVRIRALLCCIAMAAMLLSSYQQGQAEASYMPWPPATPPPPAAAAANSTSTAAANNSSSSSSTTAPPQQPTAFPMYGVTPGSLRPQECGGRCAYRCSATAYRKPCMFFCQKCCASCLCVLPGTYGNKQSCPCYNDWKTKRGGPKCP

OsGASA5

MASMAKSLLCISLVAILLLVETTAPHGQAYAIDCGAKCGYRCSKSGRPKMCLRACGTCCQRCGCVPPGTSGNENVCPCYANMTTHNGRHKCP

OsGASA6

MASSTKIPFLLLAVLLLLSIAFPSEVMAGGRGRGGGGGGGVAGGGNLRPWECSPKCAGRCSNTQYKKACLTFCNKCCAKCLCVPPGTYGNKGACPCYNNWKTKEGGPKCP

OsGASA7

MASSGSKSKTNNGFFVPSPATAMAPCFLLLLIFFFLHVDASAAAASSSSHPQLQVQQMQVKRARSLLQAPKIDCQGTCSGRCANNWKKEMCNKMCNVCCNRCNCVPPGSGQDTRHLCPCYDTMVNPHNGKLKCP

OsGASA8

MRVPPLRATTALLATLLVAASFQDLTVAADGGGGVVPVPDSVCDAKCQKRCSLKVAGRCMGLCKMCCHDCGGCVPSGPYASKDECPCYRDMVSPKSRRPKCP

OsGASA9

MALAGRLLVLFAIALLAISIAEHKALAKGSTSEHDDNVYQVSKGGQGSLKSYQCSPQCAYRCSQTQYKKPCLFFCNKCCNACLCVPSGLYGNKGECPCYNNWKTKRGGPKCP

OsGASA10

MDPASRSLSIIFFLVAVTFVVEVSGQKNEAVYHLFGGEGSLTKNECPGKCSYRCSATSHTTVCMTYCNYCCERCLCVPSGTYGNKEECPCYNNMKTQEGKPKCP

***Vitis vinifera***

VvGASA1

MGIRVLLLVTIMLFCIAEASSDDNNIFEHQHNQAVKGSAGRRLLPFLDCGGLCKERCSLHSRPNVCTRACGTCCVRCKCVPPGTYGNREMCGTCYTEMTTHGNKPKCP

VvGASA2

MKHLFPTLLLLSLLLHSCFSQPTTDGAGFCGLKCSKRCSQAAVLDRCMKYCGICCQECKCVPSGTYGNKHECPCYRDKKNSKGKPKCP

VvGASA3

MPAPMKGFCDSKCGVRCANAGVYDRCVKYCGICCQECKCVPSGTYGNKSECPCYRDKLNSKGKPKCP

VvGASA4

MLSLSMMLLLLVQNNATITEAPTPQPQQSTNGFPMHGVTQGSLHPQECAPRCTTRCSKTAYKKPCMFFCQKCCAKCLCVPPGTYGNKQFCPCYNNWKTKRGGPKCP

VvGASA5

MDLFIGHLTEFHGNLSSILDLILEREKQVKKTELHRYLSPILNHILERGVNYLQLLTPGLFFSPCSAMALKILLLLLASYLLVTKRVSANDEEFGVQATYAKAPVPAPVKTPIPAPPVKPPTVTPNPPAPVVKPPTVVPKPPAPPVSPPTVAPKPPAPVVPKPPTPPANPPTVAPKPPAPPVKPPTVVPKPPSPPVNPPTPKPPASPVKPPTVAPKPPVPPVTPPTTAPMPPVRARLDCIPLCDQRCKAHSRKNICVRACMTCCDRCKCVPPGTYGNREKCGKCYTDMTTHGNKPKCP

VvGASA6

MAKVFALFLLALLAISMLHTTVLASHGHGGHHYDQKNYGPGSLKSFQCPSQCSRRCGKTQYHKPCMFFCQKCCKKCLCVPPGYYGNKAVCPCYNNWKTKEGGPKCP

VvGASA7

MAKLVPLLLLALFSISMVATKVMAKEAQYHLDSGSYGPGSLKSNQCPSQCTRRCSKTQYHKPCMFFCQKCCAKCLCVPPGYYGNKAVCPCYNNWKTKEGGPKCP

VvGASA8

MATLRLLLVFVALLVFLAQISSDFNIEGEEMPFVSQVVVRGGNRRLMQDIDCGGLCKDRCSLHSRPNVCVRACGTCCVRCKCVPPGTSGNRELCGKCYTDMTTHGNKTKCP

VvGASA9

MKPLLATFLLVFLVLSSSFVQNAMAGSSFCDSKCAARCSKAGMKDRCLKYCGICCEECKCVPSGTYGNKHECPCYKDKKNSKGQPKCP

VvGASA10

MAISKTLIASLLISLLVYQITEAATTSGDGASSPTEKMDCGGACSARCRLSSRPNLCNRACGTCCARCNCVPPGTSGNQEICPCYANMTTRGNERKCP

VvGASA11

MKLFSVFIISILLLQAFAEASLVISNAEHSLTSVDESRDEVALHKKSHPRKINCSYACSRRCRKASRKNVCSRACKTCCKRCHCVPPGTYGNKNMCPCYASLKTHGHKPKCP

VvGASA12

MQVLFNSIKLYKLSDCKSKCAYRCSKAGWHKLCLRACNTCCERCNCVPPGTAGNEDVCPCYAKMTTHGGRHKCP

VvGASA13

MTYIYFLVSCKYRCSDTQYRNACLEFCNLCCKKCLCVPSGTYGHKEECPCYNNWKTKEGGPKCP

VvGASA14

MWVLGCKFLTYISKKKVSKMAQSSNLQSIFLLLLVAFMLLVDVSIVKCPKACNYRCSDTQYLNACLEFCNLCCQKCLCVPSGTYGHKEECPCYNNWKTKEGGPKCP

***Nicotiana tabacum***

NtGASA1

MKSNPFKLIKYFMKSNFIDLHYFIHMKILNFSCYTSNAISEAAYSYPKIDCGGACKARCRLSSRPRLCKRACGTCCARCNCVPPGTSGNTETCPCYANMTTHGNRRKCP

NtGASA2

MAISKTLFVSLVLSLLLLDQVQSIQTDQVTSNAISEAAYSYPKIDCGGACKARCRLSSRPRLCKRACGTCCARCNCVPPGTSGNTETCPCYANMTTHGNRRKCP

NtGASA3

MAISKLILVAMVFFSLLVLHLVEADNQLVVNTDATESFYTPKLDCGAACEARCRLASRQKICKRACGTCCGRCNCVPPGTSGNQELCPCYFAMTTHGGKRKCP

NtGASA4

MAISKLILVAMVFFSLLVVHLVEADNQVVVNKDATKSSYTPKLDCGAACEARCRLASRQKICKRACGTCCARCNCAPPGTSGNLELCPCYFAMTTHGGKRKCP

NtGASA5

MDISKSKALIVSLIFLLFLLSAAENHELSSSNNIDAISKALKARPNYTINCGKECTRRCKLASRQKMCMRACGTCCARCNCVPPGTSGNENICPCYSTMTTHGNRRKCP

NtGASA6

MPIKRPALPHFNHIILNPQENMKFFTLVFIAILLIQVFTEAVSINNAEDTAAQIEKAGNDGALFKKSHHHPIRKINCGYACARRCRKSSRKNVCKRACKSCCARCHCVPRGTYGNKEACPCYARLKTHGNRPKCP

NtGASA7

MKIFTLVFIAILLIQVFAEAVSFNNAEDTAAQIEKAGNEGTLFKKIHHQPIRKINCGHACARRCRKSSRKNVCKRACKSCCARCHCVPPGTYGNKEACPCYARLKTHGNRPKCP

NtGASA8

MAFQKAFAALLIASLVLVHFTHALQQGNNSKPPAPSPQAPKPLGNITFTTYIFPPRLIYNHITIILNSLTLNSEFDYVADCTGACEYRCSESSRPNLCNRACGSCCRTCHCVPPGTSGNYEACPCYFNLTTHNDTRKCP

NtGASA9

MAMAIRLVFVMALLLLFLGVKAEVSLTDPKVEEDKSQHFGLSQAFRVFTRGANRRLVQGVVLKLVKYLNNGDLAVAPAPAPHPSQLDCGGLCKYRCSLHSRPKVCIRACGTCCLRCKCVPPGTFGNREMCGKCYTEMTTHGNKTKCP

NtGASA10

ARQHFDASFLAKPPVGPTTCPVPTAQCSSACDQRCSATSHKNNCLMFCNMCCNWCQCVPPGTFGQKECCSCYNDWKTEQGTPKCP

NtGASA11

MAHSSLFECSSACDQRCSATSHKNNCLMFCNICCNWCQCVPPGTFGQKECCSCYNDWKTEQGTPKCP

NtGASA12

MAYNARLLFLSMFLVLITFSNVVEGYKKLRPEDCEPKCKYRCSATSHKKPCLFFCKKCCAKCLCVPPGTHGNKETCPCYNNWKTKEGGPKCP

NtGASA13

MEKMPCLMLLPLLIIMLLLVGTHAKITESPAPQPQPPNTFPMNGTTPGSLHPQECLPRCTYRCSKTQYKKPCMFFCQKCCAKCFCVPPGTYGNKQFCPCYNNWKTKRGGPKCP

NtGASA14

MKLCFATLLVVTLVLTSSFIQTTVAGSDFCDSKCKIRCSKAGRQDRCLKYCGICCNECQCVPSGTYGNKDECPCYRDKKNSKGKPKCP

NtGASA15

MKLLLATLLLFTLVLTPSFIQTTMAGSSYCDSKCKLRCAKAGVMDRCLKYCGICCEECKCVPSGTYGNKHECPCYRDKKNNKGKPKCP

NtGASA16

MKRIFVAFMLVFALLLTSSFLETATAKSVYCARKCKARCSKAGVKDRCVKYCELCCAKCKCVPTGTYGNKHQCPCYRDMKNFKGKPKCP

NtGASA17

SCDSKCAVRRGKAGIAKRCLTYCGIYCNKCNCVPSGNYGNKSECPCYRDMLNSKGKSKCP

NtGASA18

MKLAMITLLIVSLVLTSSFLQSAVAYDDPSSCDSKCAVRCGKAGIKKRCLTYCGICCNKCNCVPSGNYGNKSECHCYRDMLNSKGKSKCP
