# Supplementary material for: Genome-Wide Identification and Characterization of GASA Gene Family in Nicotiana tabacum
Source: Front Genet. 2022 Feb 1;12:768942. doi: 10.3389/fgene.2021.768942 (PMC8844377; doi:10.3389/fgene.2021.768942)
Supplement: Supplementary file 2 [file Table2.docx]

Table S2 collinear GASA genes among tobacco, rice, grapevine and *Arabidopsis*

| tobacco and rice | |
| --- | --- |
| *NtGASA16* | *OsGASA2* |
| *NtGASA18* | *OsGASA2* |

| tobacco and *Arabidopsis* | |
| --- | --- |
| *NtGASA3* | *AtGASA1* |
| *NtGASA4* | *AtGASA1* |
| *NtGASA5* | *AtGASA1* |
| *NtGASA16* | *AtGASA7* |

| tobacco and grapevine | |
| --- | --- |
| *NtGASA2* | *VvGASA10* |
| *NtGASA3* | *VvGASA10* |
| *NtGASA4* | *VvGASA10* |
| *NtGASA5* | *VvGASA10* |
| *NtGASA6* | *VvGASA11* |
| *NtGASA7* | *VvGASA11* |
| *NtGASA9* | *VvGASA1* |
| *NtGASA9* | *VvGASA5* |
| *NtGASA9* | *VvGASA8* |
| *NtGASA13* | *VvGASA4* |
| *NtGASA15* | *VvGASA2* |
| *NtGASA15* | *VvGASA3* |
| *NtGASA15* | *VvGASA9* |
| *NtGASA16* | *VvGASA2* |
| *NtGASA16* | *VvGASA3* |
| *NtGASA16* | *VvGASA9* |
| *NtGASA17* | *VvGASA3* |
| *NtGASA18* | *VvGASA2* |
| *NtGASA18* | *VvGASA3* |
