# Supplementary material for: Genome-Wide Identification and Characterization of GASA Gene Family in Nicotiana tabacum
Source: Front Genet. 2022 Feb 1;12:768942. doi: 10.3389/fgene.2021.768942 (PMC8844377; doi:10.3389/fgene.2021.768942)
Supplement: Supplementary file 3 [file Table3.docx]

Table S3 Primers used in this study

| List of qRT-PCR primers used in this study | |
| --- | --- |
| primer name | sequence(5'-3') |
| NtGASA1-F | CGCTTATTCCTACCCGAAAATTG |
| NtGASA1-R | TCTTACACAGTCTTGGCCTTG |
| NtGASA2-F | GGAGCATGTAAAGCAAGGTG |
| NtGASA2-R | CAAGGGCAAGTTTCAGTGTTG |
| NtGASA3-F | TGAACACGGATGCAACAGAG |
| NtGASA3-R | ACAGTTACAACGGCCACAG |
| NtGASA4-F | CGTCGAGGCAGAAAATATGC |
| NtGASA4-R | TCATGGCAAAGTAGCAGGG |
| NtGASA5-F | GCACTCAAAGCTCGTCCTAA |
| NtGASA5-R | GCTCTCATGCACATTTTCTGTC |
| NtGASA6-F | GTTTTCACTGAGGCTGTTTCC |
| NtGASA6-R | GCATACCCACAGTTGATCTTCC |
| NtGASA7-F | GGAAAATCAACTGTGGGCATG |
| NtGASA7-R | GTGGAACACAATGGCATCTTG |
| NtGASA8-F | TGTGAATATAGGTGCAGCGAG |
| NtGASA8-R | GGGCAAGCTTCATAATTTCCAG |
| NtGASA9-F | TGGATTGTGGAGGGTTATGC |
| NtGASA9-R | TCTCTGTTGCCAAAGGTACC |
| NtGASA10-F | GCTCCGCAACATCACATAAG |
| NtGASA10-R | TCTTCCAATCGTTGTAGCAGG |
| NtGASA11-F | GTCTCTTTGAATGTTCGAGTGC |
| NtGASA11-R | CACATTGGCACCAGTTACAAC |
| NtGASA12-F | AACTCCGTCCTGAAGATTGC |
| NtGASA12-R | TGTTGCCATGAGTTCCAGG |
| NtGASA13-F | GCTTGATGCTACTCCCTCTTC |
| NtGASA13-R | CTGAGGTGCTGGAGATTCTG |
| NtGASA14-F | TGTTTGAAGTATTGTGGGATATGTTG |
| NtGASA14-R | AGGGCACTCGTCTTTGTTC |
| NtGASA15-F | TGTTCACTCTTGTTCTTACCCC |
| NtGASA15-R | GCATTCTTCACAACAAATTCCAC |
| NtGASA16-F | CCTTTCTTGAAACCGCAACG |
| NtGASA16-R | GATCCTTCACTCCTGCCTTG |
| NtGASA17-F | CAGTGAGGCGTGGGAAAG |
| NtGASA17-R | AGCATGTCTCTATAACAAGGGC |
| NtGASA18-F | GTTCATGCGATTCTAAGTGCG |
| NtGASA18-R | TCCACAGTAGGTCAAGCAAC |
